# The Role of von Willebrand Factor in Vascular Inflammation: From Pathogenesis to Targeted Therapy

**DOI:** 10.1155/2017/5620314

**Published:** 2017-05-28

**Authors:** Felice Gragnano, Simona Sperlongano, Enrica Golia, Francesco Natale, Renatomaria Bianchi, Mario Crisci, Fabio Fimiani, Ivana Pariggiano, Vincenzo Diana, Andreina Carbone, Arturo Cesaro, Claudia Concilio, Giuseppe Limongelli, Mariagiovanna Russo, Paolo Calabrò

**Affiliations:** Division of Cardiology, Department of Cardio-Thoracic and Respiratory Sciences, University of Campania “Luigi Vanvitelli” and A.O. dei Colli Monaldi Hospital, Naples, Italy

## Abstract

Beyond its role in hemostasis, von Willebrand factor (VWF) is an emerging mediator of vascular inflammation. Recent studies highlight the involvement of VWF and its regulator, ADAMTS13, in mechanisms that underlie vascular inflammation and immunothrombosis, like leukocyte rolling, adhesion, and extravasation; vascular permeability; ischemia/reperfusion injury; complements activation; and NETosis. The VWF/ADAMTS13 axis is implicated in the pathogenesis of atherosclerosis, promoting plaque formation and inflammation through macrophage and neutrophil recruitment in inflamed lesions. Moreover, VWF and ADAMTS13 have been recently proposed as prognostic biomarkers in cardiovascular, metabolic, and inflammatory diseases, such as diabetes, stroke, myocardial infarction, and sepsis. All these features make VWF an attractive therapeutic target in thromboinflammation. Several lines of research have recently investigated “tailor-made” inhibitors of VWF. Results from animal models and clinical studies support the potent anti-inflammatory and antithrombotic effect of VWF antagonism, providing reassuring data on its safety profile. This review describes the role of VWF in vascular inflammation “from bench to bedside” and provides an updated overview of the drugs that can directly interfere with the VWF/ADAMTS13 axis.

## 1. Historical Perspective

The story of von Willebrand factor starts in Finland in the first years of the 1900s. Professor Erik Adolf von Willebrand was an internist at the University of Helsinki interested in genetic and blood coagulation, and his studies led him to uncover a “new form of hemophilia”, von Willebrand disease (VWD), the most common inherited bleeding disorder [1]. In 1925, he firstly examined Hjiordis, a 7-year-old girl from Föglö (Aland archipelago, Finland) who suffered from frequent and remarkable episodes of bleeding from the nose and lips following tooth extraction. He immediately noticed that joint bleeding, common in hemophilia, was rare. At the age of 13 years, Hjiordis died for a fatal bleeding during her fourth menstrual period. von Willebrand also mapped the family pedigree: both of her parents came from families with bleeding disorders, and all but two of her 12 siblings had bleeding symptoms (4 of them experienced fatal bleeding) [2]. In the ‘70s, several studies showed how factor VIII, impaired in hemophilia A, was not responsible for this hereditary disease. A newly discovered protein was recognized as the cause of hemorrhagic diathesis in these patients: the von Willebrand factor (VWF) [3, 4].

## 2. VWF Architecture

VWF is a large plasma adhesive glycoprotein with multimeric structure [5], selectively produced in megakaryocytes (MKs) and endothelial cells (ECs). Encoded on the short arm of chromosome 12, the basic monomer of VWF consists of 2.050 residues and contains four repeated domains assembled in the following order: D1-D2-D'-D3-A1-A2-A3-D4-C1-C2-C3-C4-C5-C6-CK [6]. A1 and A3 domains are mainly involved in thrombosis: A1 binds to GpIb*α* on the platelet surface and microfibrillar collagen (type VI); A3 binds to fibrillar collagens (types I and III) [7]. VWF multimerization is a multistep process: monomers of VWF firstly dimerize in the endoplasmic reticulum (ER); then, they link in mature VWF multimers or “concatemers” in the Golgi and post-Golgi compartments [7, 8]. Mature multimers of VWF are packaged in helicoidally structures and stored in Weibel-Palade bodies (WPBs) in ECs and *α*-granules in MKs and platelets [7]. VWF molecules are produced and secreted with different sizes, ranging from dimers to ultralarge (UL) multimers (up to 100 monomers). In ECs, the release of VWF from WPBs occurs in both constitutive and regulated manner. On the other hand, platelet activation is necessary for VWF secretion from *α*-granules and no constitutive release has been reported [9]. Ultralarge (UL) multimers of VWF (UL-VWF) are extremely reactive and hyperadhesive, prone to interact with platelets causing spontaneous platelet adhesion/aggregation. To avoid the accumulation of UL-VWF multimers, protein size is finely regulated by a metalloprotease, ADAMTS13 (a disintegrin and metalloprotease with thrombospondin motif). Under flow condition, ADAMTS13 binds to the A2 domain and, acting as enzymatic scissors, cleaves VWF “long-chain” multimers, and releases smaller, less active VWF molecules. The last tangle up in a globular quiescent conformation, hiding the A1 and A2 domains, involved, respectively, in VWF interaction with platelet and ADAMTS13 [10].

## 3. Pathophysiology of VWF in the Bloodstream

In intact vessels, at low-shear conditions, VWF circulates in a globular shape, as a “folded spring”. However, VWF is highly dynamic in the bloodstream, and at high shear rates (above a threshold of 5000 s^−1^), globular-shaped VWF rapidly unfolds and elongates into a long-chain conformation, changing its functional status from inactive to highly reactive [11]. VWF interacts with platelets via two receptors: glycoprotein (Gp) Ib-IX-V complex and integrin *α*IIb*β*3 [12]. In injured vessels, the VWF-GpIb interaction enables platelets to roll on damaged ECs and subendothelium and to establish a preliminary, unstable adhesive interaction. This low-affinity interaction gives time for the activation of the *α*IIb*β*3 integrin that in turn binds to VWF, fibrinogen, fibrin, and different ECM (extracellular matrix) proteins leading to a more firm platelet adhesion and aggregation [7]. The role of VWF in platelet adhesion and aggregation is crucial under high-shear conditions (i.e., arterioles, microcirculation, and critical artery stenosis): at increasing shear rates (above 1000 s^−1^), aggregate formation is progressively more VWF-dependent. As a result, at very high shear rates (above 10,000 s^−1^), thrombus formation is almost exclusively mediated by the VWF-GpIb interaction [12]. VWF also participates indirectly in the coagulation cascade binding to factor VIII (FVIII). This interaction protects FVIII from proteolytic clearance prolonging its half-life and also improves its function directing its localization at the site of vascular injury [13]. Plasma level of VWF is determined by both genetic [14, 15] and nongenetic factors, such as ABO blood group, aging, and sexual hormones (i.e., estrogens). Endothelial dysfunction and activation, vascular aging, and arterial stiffness are all associated with increased levels of VWF [16, 17]. Nitric oxide (NO), a marker of endothelial health, exerts an inhibitory effect on VWF release by ECs, probably blocking the granule membrane fusion process or inhibiting calcium mobilization from WPBs [15, 18, 19]. Endothelial dysfunction probably represents the background that links VWF, inflammation, and thrombosis [18–20].

## 4. VWF in Vascular Inflammation

**Figure 1:**
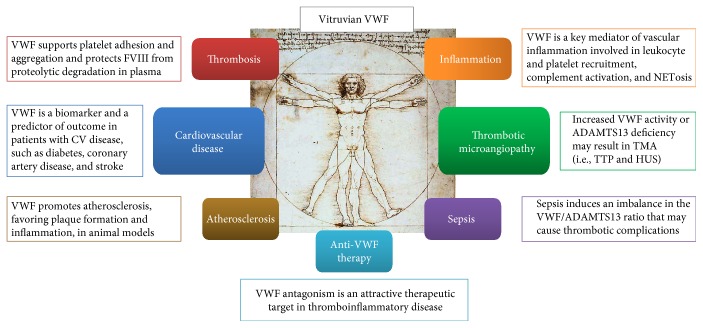
Functional heterogeneity of von Willebrand factor (VWF). VWF is best known for its role in hemostasis and thrombosis, supporting platelet adhesion/aggregation and protecting FVIII from proteolytic degradation in blood flow. However, it is now clear that VWF functions extend much further than that. VWF plays a key role in vascular inflammation, favoring leukocyte recruitment and extravasation, activating complement cascade, and participating in NETosis. In cardiovascular disease, including CAD and stroke, VWF is a predictor of future CV events. In atherosclerosis, VWF promotes plaque formation and inflammation in animal models. An increase in VWF activity or ADAMTS13 deficiency may result in microvascular obstruction and thrombotic microangiopathy (TMA). In sepsis, inflammatory and infective stimuli may induce an acute imbalance in the VWF/ADAMTS13 ratio with possible thrombotic complications (i.e., DIC). Finally, a growing interest is emerging on selective VWF antagonism as a new therapeutic option to provide a further advance in the treatment of thrombotic and inflammatory disorders. VWF: von Willebrand factor; ADAMTS13: a disintegrin and metalloprotease with the thrombospondin motif; TMA: thrombotic microangiopathy; CV: cardiovascular; CAD: coronary artery disease; TTP: thrombotic thrombocytopenic purpura; HUS: hemolytic uremic syndrome.

**Figure 2:**
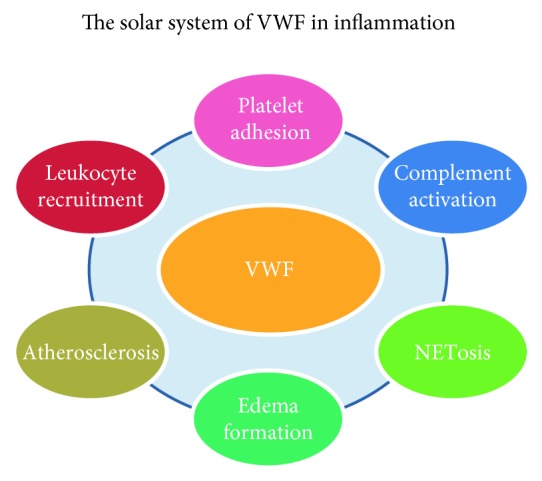
The “solar system” of VWF-mediated vascular inflammation. VWF is central in the “solar system” of vascular inflammation, and many inflammatory pathways orbit in its “gravitational field.” VWF supports leukocyte and platelet recruitment in inflamed tissue, modulates vascular permeability and edema formation, may promote atherosclerotic plaque formation and inflammation, and provides an activating surface for complement activation and NETosis. All these mechanisms may contribute to tissue injury and organ failure in thromboinflammatory disorders.

The VWF/ADAMTS13 axis exerts a pivotal role in vascular inflammation and thrombosis [21]. Thrombosis, with the recruitment of platelets to the site of vessel's injury, and immune response, with the recruitment of leukocytes in inflamed tissues, have traditionally been considered two distinct pathways. This was the “dominant scientific view” until the new concept of “immunothrombosis” was introduced [22]. Several studies suggest how thrombosis can be considered a mechanism of intravascular immunity, limiting bacteria from spreading in the bloodstream. On the other hand, systemic inflammation and uncontrolled immunity response (i.e., sepsis) can lead to the extreme “pathological” activation of thrombotic cascade and disseminated intravascular coagulation (DIC) [22]. VWF may represent a possible “connection bridge” between the hemostatic and the inflammatory pathway (Figures 1 and 2) mediating adhesion and recruitment of both platelets and leukocytes: the network of cell interactions and aggregation is becoming more intriguing [22, 23].

### 4.1. VWF Secretion in Inflammation

Like C-reactive protein [24–27], VWF is an acute phase reactant [28], and its level increases in inflammatory and metabolic disorders (i.e., glucose intolerance, diabetes, and obesity) [29, 30]. In contrast, similar to negative acute phase proteins, ADAMTS13 activity declines in patients with systemic inflammation [31]. As a result, inflammation may potentially activate thrombosis, inducing a marked imbalance between VWF and ADAMTS13. Different mediators of inflammation (cytokines, superoxide anions, histamine, and thrombin) produce an increase in VWF levels through various mechanisms [32, 33]; for example, IL-8 and TNF-*α* significantly stimulate the release of UL-VWF by ECs, whereas IL-6 inhibits the UL-VWF cleavage by ADAMTS13. These data suggest that cytokines may potentially affect VWF metabolism, and in inflammatory disease, active UL-VWF multimers may accumulate in plasma and induce a prothrombotic state. However, this fascinating hypothesis needs confirmatory studies.

### 4.2. VWF and Leukocyte Recruitment: Rolling, Adhesion, and Extravasation

**Figure 3:**
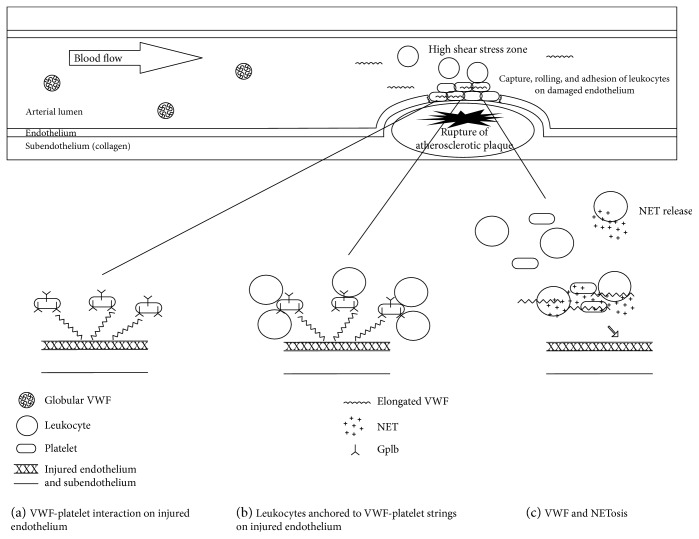
VWF-mediated thromboinflammation in high-shear conditions. At high shear rates (e.g., arterioles, microcirculation, and artery stenosis), the inactive globular-shaped VWF rapidly unfolds and elongates in a highly reactive long-chain conformation. The elongated VWF can bind to platelets (a), allowing them to roll and adhere to the damaged endothelial surface. Platelet-decorated UL-VWF strings on activated endothelium represent a solid anchoring matrix for leukocyte adhesion (b), so permitting leukocyte recruitment in the inflamed site. In the bloodstream, VWF also binds to “neutrophil extracellular traps” (NETs) (c), inflammatory mediators (decondensed nucleosomes, extracellular DNA, and proteins) released by activated neutrophils, creating a network able to recruit both platelets and leukocytes and to promote thrombus formation.

Leukocyte rolling, adhesion, and extravasation are hallmarks of inflammation [23, 34]. Initial rolling on endothelium results in a slowdown of circulating leukocytes, predominantly mediated by the interaction of selectins (P-selectin and E-selectin) on ECs and P-selectin glycoprotein ligand-1 (PSGL-1) on leukocytes [35]. This unstable bond promotes leukocyte activation and stable adhesion on EC layer mediated by the interaction between leukocyte *β*2-integrins (*α*L*β*2, *α*M*β*2, *α*D*β*2, and *α*X*β*2) and endothelial intercellular adhesion molecules (ICAMs). This more stable interaction finally allows leukocyte transendothelial migration and extravasation [36]. In both venous and arterial environment (at low and high shear rates, respectively), leukocyte adhesion and extravasation may be supported by the VWF-leukocyte interaction (Figure 3).

#### 4.2.1. VWF-Leukocyte Interaction in “Venous” Low-Shear Condition

In the '90s, initial studies demonstrated a relation between VWF and leukocyte recruitment at a low shear rate. In 1993, Wagner firstly reported that VWF indirectly regulates the expression of P-selectin on the EC surface [37]. In 2001, Denis et al. confirmed these results in models of inflamed venules and, most importantly, showed how the absence of VWF correlates with a deficient leukocyte recruitment at a low shear rate (95–100 s^−1^) [38]. In the same year, Koivunen et al. showed that leukocytes could bind to VWF through the interaction between the leukocyte-specific *β*2 integrins and the leucine–leucine–glycine (LLG) sequences contained in the A2 and D3 domains of VWF [39]. Some years later, Pendu et al. [23] clearly demonstrated that VWF could interact directly with polymorphonuclear leukocytes (PMNs), under static- and low-shear conditions (at wall shear rate: 50 s^−1^). All these results suggest that VWF may create an adhesive surface on activated ECs to capture monocytes and PMNs and mediate their stable (through *β*2-integrins) and unstable (through PSGL-1) adhesion [23, 39].

#### 4.2.2. VWF-Leukocyte Interaction in “Arterial” High-Shear Condition

The “adhesive” mechanisms described above, efficient at a low shear rate, probably change under high-shear stress conditions (i.e., arterial and arteriolar flows) where a more complex interaction among VWF, leukocytes, and platelets is necessary. Bernardo et al. [40] showed how, under high-shear stress (20–40 dynes/cm^2^), leukocytes tethered and rolled on an ideal matrix of platelets adherent to UL-VWF strings on activated ECs, but not directly on the endothelial layer. In this model, platelet-decorated UL-VWF strings represent a firm anchor for leukocyte adhesion. Authors speculate that this mechanism may support the accumulation of leukocytes in inflamed vascular lesions, such as arterial atherosclerotic plaques.

#### 4.2.3. VWF and Leukocyte Extravasation

Beyond rolling and adhesion, VWF may also contribute to the extravasation of leukocytes in inflamed tissues. In a murine model of thioglycollate-induced peritonitis, Petri et al. [41] showed how VWF mediates PMN extravasation, modulating endothelial barrier permeability. Also, in their model intravenous injection of anti-VWF or anti-GpIb antibodies inhibited PMN recruitment across the inflamed endothelium, demonstrating that VWF-mediated PMN extravasation is strictly dependent on the presence of platelets and GpIb. Looking at their results, the authors suggest that in the inflamed vessel, platelet interaction with VWF anchored on activated ECs could promote the opening of endothelial junctions and thereby facilitates the leukocyte diapedesis process.

### 4.3. VWF and Complement System

The complement system is an enzyme cascade with a defensive role against infection, diffusely involved in the pathogenesis of inflammatory and thrombotic disorders (i.e., autoimmune diseases, ischemia-reperfusion injury, hemolytic uremic syndrome [HUS], and thrombocytopenic purpura [TTP]) [42]. Three complement pathways have been described: classical, alternative, and lectin. They all converge into a final common generation of the membrane attack complex (MAC), the cytotoxic component of the complement system [42]. Recent evidence suggests that VWF may promote the activation of alternative complement pathway (AP). In experimental models of the human umbilical vein ECs (HUVECs) [43], complement products have been detected to be attached on VWF strings. VWF multimers anchored on ECs create an “activating surface” for AP: complement compounds may bind to the EC-anchored VWF strings and promote cascade activation [43] leading to the final formation of the MAC. In thrombotic microangiopathies (TMA) (i.e., TTP and HUS), ADAMTS13 deficiency has been associated with abnormal complement activation [44]. As known, many patients experienced TTP and HUS episodes during inflammatory conditions (i.e., infections) [45]. In these view, both ADAMTS13 deficiency and inflammatory stimuli may increase the concentration of UL-VWF strings on ECs and, in turn, trigger AP activation, with an uncontrolled synthesis of anaphylatoxins/MAC that aggravates tissue injury [43, 44, 46].

### 4.4. VWF, NETosis, and HNPs: Thrombosis and Host Defense

Neutrophils are principal actors in immunothrombosis [47] and have two points of contact with the VWF system: human neutrophil peptides (HNPs) and NETosis. HNPs, also known as *α*-defensins, are small cationic antimicrobial peptides released from activated neutrophils, involved as mediators of vascular inflammation in metabolic and inflammatory disorders (including acute coronary syndrome [ACS]) [48]. In 2016, Pillai et al. [49] demonstrated an inhibitory effect of HPNs on ADAMTS13 proteolytic activity and an increased level of HPNs in patients with acquired TTP. NETosis is a recently discovered cell death process that links sterile inflammation and thrombosis [50]. In inflammatory condition, activated neutrophils release “neutrophil extracellular traps” (NETs): inflammatory mediators with a cytotoxic effect, formed by decondensed nucleosomes, extracellular DNA, and proteins derived from intracellular granules, such as neutrophil elastase (NE) and myeloperoxidase (MPO) [51]. VWF can bind to NETs [52], and the VWF-NET network may mediate both leukocyte and platelet recruitment in high-shear conditions (Figure 3). Interestingly, heparin may interfere with DNA-VWF binding and blocks leukocyte adhesion. In this view, the VWF-NET interaction could promote vascular inflammation and may be a potential target for combined anti-inflammatory and antithrombotic therapies [52–54].

## 5. VWF in Atherogenesis

The role of VWF in atherogenesis is still unclear. In the ECs of the atherosclerotic lesion, there is a high concentration of WPBs (in which the VWF in stored) [55] and oxidized LDLs and high-shear stress, two factors involved in atherosclerosis, can induce WPB exocytosis [56, 57]. In the ‘70s, Fuster et al. observed that homozygous VWF-deficient (VWF −/−) pigs hardly developed aortic atherosclerosis whereas VWF +/+ pigs showed significant atherosclerosis [58]. In 2001, Methia et al. demonstrated that early atherosclerotic lesions, fatty streak and early fibrous plaques, formed in the aortic sinus of VWF −/− mice were smaller and contained fewer macrophages than those in VWF +/+ mice. Moreover, in VWF +/+ mice, atherosclerotic lesions were mostly located at the branch points of the arteries (known as regions of disturbed flow), whereas they were not so prominent in these areas in mice with VWF deficiency [59]. In 2003, Qin et al. proved in vitro that VWF directly stimulates the proliferation of smooth muscular cells (SMCs), one of the major constituents of atherosclerotic plaques, in a direct dose-dependent way and that low-shear stress in vivo accelerates intimal hyperplasia proportional to VWF expression [60]. Also, ADAMTS13 indirectly modulates atherosclerosis by cleaving UL-VWF multimers that may actively participate in the macrophage and neutrophil recruitment in inflamed plaques, with a potential protective effect on atherosclerotic lesion progression [61]. Finally, it has been observed in animal models that anti-VWF agents have a protective effect against atherogenesis [62]. Molecules that disturb the interaction between VWF and GpIb, like monoclonal antibodies AJvW-2 [62] and AJW200 [63], the VWF recombinant fragment VCL [64], and ATA (aurintricarboxylic acid) [65] have shown to prevent neointima formation and growth, by inhibiting platelet adhesion to the vessel wall and SMC proliferation. The link between VWF and atherogenesis has been recently enriched by recent studies on the interaction between high-density lipoprotein (HDL), apolipoprotein-AI (ApoA-I), and VWF. As a principal actor in reverse cholesterol transport (RCT), HDL has antiatherogenic proprieties. Chung et al. recently demonstrated how HDL/ApoA-I can exert an anti-VWF effect reducing VWF secretion, preventing self-association of hyperactive UL-VWF multimers, and interfering with the capacity of VWF to bind to the vessel wall [66]. Although this evidence is in animal models, an unequivocal protective role of VWF deficiency in atherosclerosis has not been demonstrated in humans. Indeed, autopsy studies showed that even patients with congenital VWD type 3 (complete VWF deficiency) were not fully protected from atherosclerosis [67]. Therefore, additional studies are needed to clarify VWF's real role in atherosclerosis.

## 6. VWF in Metabolic and Cardiovascular Disease: Animal Models and Clinical Evidence

### 6.1. Animal Models

The role of VWF in stroke has been diffusely investigated in animal models. In mouse models of ischemic stroke, VWF activates thromboinflammatory pathways involved in postischemic inflammatory response and ischemia/reperfusion (I/R) injury, and complete deficiency of VWF is protective for cerebral infarction [68, 69]. Conversely, mice lacking ADAMTS13 have enhanced inflammatory response following I/R brain injury, increased infarct volume, and more severe neurological deficits compared with wild-type (WT) mice [68, 70]. ADAMTS13 −/− mice [70] also exhibited enhanced neutrophil infiltration in the infarcted and peri-infarcted region and increased activity of the inflammatory markers (such as MPO, TNF-*α*, and IL-6). This enhanced acute inflammatory response in mice lacking ADAMTS13 has been shown to be entirely mediated by VWF [70]. Similarly, several studies on mice suggest an involvement of the VWF/ADAMTS13 axis in myocardial ischemia/reperfusion injury [71, 72]. Finally, VWF could aggravate I/R injury through its capacity of promoting complement activation [73, 74].

### 6.2. Clinical Studies

VWF and ADAMTS13 have been proposed as useful biomarkers and predictors of prognosis in patients with cardiovascular and metabolic disease [75]. In diabetic patients, VWF and ADAMTS13 activities correlate with CV outcome and risk of chronic complications and may predict a response to therapies [30, 76]. In the Rotterdam study, elevated VWF levels predict the highest risk of ischemic stroke in general population [77]. Conversely, patients with VWD have shown a reduced risk of ischemic stroke [78]. Furthermore, in patients with chronic cerebrovascular disease, VWF levels are higher than those in healthy individuals, but lower than those in acute ischaemic stroke/TIA patients, suggesting a crescent gradient of VWF activity among normal, chronic, and acute cerebrovascular conditions [79]. In this view, VWF may become a hopeful target in stroke management [21]. Recent evidence also suggests a clinical relation between VWF and coronary artery disease. In the European Concerted Action on Thrombosis and Disabilities (ECAT) study [80], in stable patients with angiographically documented coronary artery disease (CAD), higher levels of VWF:antigen (VWF:Ag) were independently associated with an increased incidence of MI and sudden death. More recently, in the ATHEROREMO-IVUS study [81], in patients with stable CAD (SCAD), an increased level of VWF:Ag was associated with higher coronary plaque burden, adverse CV outcome, and death during 1-year of follow-up. VWF is also an independent risk factor for first STEMI: levels of VWF are significantly increased in patients with first ST-elevation myocardial infarction (STEMI) rather than in controls [82]. Finally, Marcucci et al. reported a possible relation between lower levels of ADAMTS13 and higher residual platelet reactivity (RPR), a marker of resistance to antiplatelet therapy associated with increased risk of ischemic events [83]. These results confirm a cross-talk between thrombosis and inflammation suggesting that the VWF and ADAMTS13 may influence both clinical outcome and response to therapy in metabolic and cardiovascular disease.

## 7. VWF, TMA, and Infective Disease: Cross-Talk between Infection and Thrombosis

Dysfunctional activation of the VWF/ADAMTS13 system in thrombotic microangiopathies (TMAs) (such as TTP, HUS, and DIC) results in microvessel occlusion by a complex web of VWF-rich thrombi causing tissue hypoperfusion and organ failure, potentially life-threatening [84]. As known, TMAs are possible complications of infective disease and sepsis [45]. In infective disease, the presence of inflammatory stimuli may trigger an acute imbalance in the VWF/ADAMTS13 system through an increase in VWF multimer secretion and an inhibition of ADAMTS13 activity [85] and consequently lead to microvascular occlusion and multiorgan failure. In this view, it is interesting to observe how in clinical studies on patients with SIRS and sepsis, VWF and ADAMTS13 have shown to be a prognostic biomarker identifying patients with worse outcome and a higher risk of death [86–88]. The ADAMTS13 pattern also differed in infective (sepsis) or noninfective SIRS [89]: septic patients have lower levels of ADAMTS13 than patients with noninfectious SIRS. These studies together bring new lights on the link between thrombosis, inflammation, and infective diseases. Further clinical studies are needed to clarify if restoration of normal activity levels of ADAMTS13 (i.e., with plasma or recombinant ADAMTS13 infusions) may neutralize VWF-system prevalence and prevent microvascular occlusion and organ failure in patients with SIRS and sepsis.

## 8. VWF in Vasculitis

A recent study in a model of vascular inflammation provides food for thoughts in VWF research. Hillgruber et al. [90] described the impact of VWF on in leukocytoclastic vasculitis (LcV), an immune complex- (IC-) mediated vasculitis (ICV) common in dermatology, caused by the precipitation of IC in the vessel wall and subsequent recruitment of neutrophils. Compared with those of healthy skin controls, a massive accumulation of VWF was present in skin biopsies of patients suffering from LcV. This result was also confirmed in experimental murine models of ICV, identifying ICs as potent VWF secretagogues in ECs. Also, VWF promoted leukocyte recruitment and edema formation, probably regulating endothelial permeability [90, 91]. To test anti-inflammatory properties of anti-VWF therapy, Hillgruber et al. used polyclonal anti-VWF antibodies able to block epitopes in the D'-D3 region (that mediate the VWF/leukocyte interaction) without interfering with the GpIba-VWF interaction. Interestingly, anti-VWF treatment showed a vascular anti-inflammatory effect blocking leukocyte recruitment and edema formation and was useful in both prophylactic and therapeutic administrations. Of note, no interference with hemostasis was registered. This result confirms VWF antagonism as a promising target therapy in inflammatory vascular disorders.

## 9. Anti-VWF Target Therapy in Thromboinflammation

Targeting the VWF/ADMTS13 pathway in thromboinflammation with specific drugs is a fascinating hypothesis (Table 1).

### 9.1. Nonspecific Anti-VWF Therapy

Daily-used drugs have demonstrated a VWF-modulating activity in different clinical conditions. Anti-inflammatory agents such as corticosteroids [92] or TNF*α* inhibitors [93] block the release of acute phase reactants, including VWF. Colchicine, an alkaloid with anti-inflammatory effects, inhibits VWF release inducing microtubule disruption [32]. Statin, lipid-lowering medications with anti-inflammatory effects, recently demonstrated to significantly reduce plasma levels of VWF [94]. Low-molecular weight heparins (LMWHs) can, directly and indirectly, antagonize VWF activity through antithrombotic and anti-inflammatory mechanisms [95–97]. N-Acetylcysteine (NAC) is an important antioxidant with anti-inflammatory properties [98]. Recent findings highlighted the ability of NAC to exert a direct negative modulation of VWF, mimicking ADAMTS13 activity, degrading UL-VWF multimers, and inhibiting VWF cell interaction (with platelets and leukocytes) by the disruption of the disulfide bond in the VWF A1 domain [99]. Several studies also suggest the benefit of NAC in patients with severe TTP crisis [100] probably blocking both proinflammatory and prothrombotic effects of VWF. In summary, unselective VWF antagonism of daily-used drugs may play beneficial in inflammatory and thrombotic disorders. However, data are not sufficient, and the beneficial effect of the anti-VWF action is hard to quantify in terms of benefit/risk ratio. To clarify the potential benefit of VWF antagonism, specific drugs need to be tested in preclinical and clinical studies.

### 9.2. Specific Anti-VWF Therapy

Specific VWF antagonism represents a new interesting issue in thromboinflammation therapy with a potential role in metabolic and cardiovascular disease. Leukocyte adhesion and extravasation, vascular permeability, edema formation, abnormal complement activation, ischemia-reperfusion injury, NETosis, and inflammatory-induced microvascular thrombosis are all potential therapeutic targets. Experimental models suggest that the neutralization of VWF activity using specific pharmacological compounds could result in both antithrombotic and anti-inflammatory effects [71, 101]. Recently, different classes of drugs (including antibodies, nanobodies, and aptamers) have been tested in preclinical and clinical studies. Further studies are needed for clinical approval.

#### 9.2.1. Antibodies

Anti-VWF antibodies have been widely tested in animal models. Pendu et al. demonstrated how the use of specific antibodies directed to the D'D3 region permits to selectively block VWF function in vascular inflammation, without interfering with its hemostatic function [23]. Hillgruber et al. [90] used polyclonal VWF-directed antibodies to target the D'D3 region in models of cutaneous inflammation inducing an immediate regression of inflammatory response and a significant reduction in leukocyte recruitment and vascular permeability. Also in this case, the anti-inflammatory effect did not interfere with hemostasis [90]. Monoclonal antibodies have also been tested in animal models of cardiovascular disease (coronary thrombosis, stroke, and in-stent stenosis): GPG-290, 6B4-Fab, h6B4-Fab, and AJW200 directly target the VWF-GpIb binding; 82D6A3 blocks VWF-collagen interaction; and finally, SZ-123 inhibits both mechanisms [102–109]. All compounds demonstrated a powerful antithrombotic effect and possible anti-inflammatory proprieties [102] with no important side effects, in terms of spontaneous bleeding and thrombocytopenia [102–109].

#### 9.2.2. Aptamers

The aptamer is a new pharmacological class composed of small RNA/DNA molecules, of 20 to 100 nucleotides, highly specific and nonimmunogenic [110]. Two molecules have been tested in preclinical and clinical studies: ARC1779 that targets VWF A1 domain-GpIb interaction [111–113] and ARC15105 that targets A1 domain on VWF and blocks VWF-collagen binding [114, 115]. Both showed an effective inhibition of VWF activity with a significant antiplatelet effect, without causing serious adverse events [111–115], but no anti-inflammatory effect has been reported.

#### 9.2.3. Nanobodies

Nanobodies represent a novel class of highly specific therapeutic proteins with broad application prospects in research and clinical practice [116]. ALX-0081 (caplacizumab) is an anti-VWF humanized nanobody that selectively targets the A1 domain, blocking the VWF-GpIb interaction. In the TITAN trial [117], a randomized placebo-controlled phase 2 study, 75 patients with acquired TTP were randomly assigned to subcutaneous caplacizumab (10 mg daily) or placebo, as an adjunct to standard therapy. Caplacizumab induced a more rapid resolution of the acute TTP episode and more efficient organ protection, associated with an increased tendency toward mild/moderate bleeding as compared with the placebo group [117].

#### 9.2.4. Recombinant ADAMTS13 Therapy

Recombinant ADAMTS13 (rADAMTS13) therapy showed anti-inflammatory and antithrombotic effects [118] on preclinical models of cerebrovascular disease and TTP [119–123]. In mouse models of stroke treated with tissue plasminogen activator (tPA) [118, 120], rADAMTS13 was protective against ischemic brain injury, prevented inflammation-induced cerebral endothelial damage [119], and reduced tPA-associated hemorrhage, probably by regulating the blood-brain barrier (BBB) integrity [120]. These results confirm previous findings [68] reporting that infusion of rADAMTS13 reduces infarct volume and improves ischemic stroke outcome without producing cerebral hemorrhage. In mouse models of intracerebral hemorrhage (ICH) [121], treatment with rADAMTS13 reduced chemokine and cytokine level, adhesion molecule (ICAM-1) expression, metalloprotease (MMP-9) and MPO activity, and microglia activation. The protective effect of rADAMTS13 also determined a reduction in brain edema and neutrophil recruitment, with a better preservation of the BBB integrity as compared with that of the control group. Interestingly, on cultured ECs, the anti-inflammatory effect of rADAMTS13 was reversed by recombinant von Willebrand factor (rVWF), suggesting that VWF mediates the effect of ADAMTS13 on vascular inflammation. Finally, in ADAMTS13 −/− mouse models of TTP, the use of a new rADAMTS13 product (BAX930) [123] demonstrated both prophylactic and therapeutic efficacies, with a favorable preclinical profile, supporting future clinical development. Looking at recent studies, specific anti-VWF and recombinant ADAMTS13 therapies represent an appealing field of research. Initial results are encouraging, even if no definitive clinical data are available.

## 10. Conclusions

VWF is a key mediator of vascular inflammation. Recent lines of evidence suggest its role in leukocyte and platelet recruitment in inflamed tissue. In experimental models, VWF supports the activation of multiple inflammatory pathways, such as complement cascade and NETosis, promotes atherosclerosis favoring plaque progression and complication, and exacerbates ischemia/reperfusion injury. In patients with cardiovascular disease, including CAD and stroke, VWF and ADAMTS13 are both predictors of future CV events. All these findings suggest that selective VWF antagonism is an attractive therapeutic option to provide further advances in the treatment of thrombotic and inflammatory vascular disorders. In the next future, more preclinical and clinical studies are expected to throw open new avenues of investigation into the VWF/ADAMTS13 system.

## Figures and Tables

**Table 1:** VWF-targeted pharmacotherapy. Specific and nonspecific VWF antagonists and mechanism of action. VWF: von Willebrand factor; GpIb: glycoprotein Ib; Fab: fragment antigen binding; TNF: tumor necrosis factor; MA: monoclonal antibody; Ref.: references.

Drugs	Mechanism of action	Ref.
*Nonspecific VWF antagonists*		
Statins	Anti-inflammatory effect (?)	[94]
Heparin	Binds VWF-A1 domain + anti-inflammatory effect (?)	[95–97]
N-Acetylcysteine (NAC)	ADAMTS13-like activity	[98–100]
Anti-TNF*α*	Anti-inflammatory effect (?)	[93]
Corticosteroids	Anti-inflammatory effect (?)	[92]
Colchicine	Microtubule disruption	[32]
Aurintricarboxylic acid (ATA)	Inhibits VWF-GpIb interaction	[65]
*Specific VWF antagonists*		
h6B4-Fab	MA: inhibits VWF-GpIb interaction	[103]
GPG-290	MA: inhibits VWF-GpIb interaction	[104]
AJvW-2	MA: inhibits VWF-GpIb interaction	[62]
AJW200	MA: inhibits VWF-GpIb interaction	[102, 105]
82D6A3	MA: inhibits VWF-collagen interaction	[107]
SZ-123	MA: inhibits VWF interaction with GpIb and collagen	[109]
ARC1779	Aptamers: inhibits VWF-GpIb binding	[111–113]
ARC15105	Aptamers: inhibits VWF-collagen binding	[114]
ALX-0081 (caplacizumab)	Nanobody: inhibits VWF-GpIb interaction	[117]
VCL	VWF recombinant fragment	[64]
BAX930	Recombinant ADAMTS13 (rADAMTS13)	[123]

## Abbreviations

Fab: Fragment antigen binding
STEMI: ST-elevation myocardial infarction
TTP: Thrombotic thrombocytopenic purpura
UL-VWF: Ultralarge von Willebrand factor
VWF: von Willebrand factor
VWF:Ag: von Willebrand factor antigen.
